# Self-Regulation Behaviour Styles and Levels of Asthenia in Humanities Students and People living with HIV in Russia during the COVID-19 Second Wave

**DOI:** 10.1192/j.eurpsy.2026.11464

**Published:** 2026-07-31

**Authors:** V. I. Rozhdestvenskiy, V. V. Titova, I. A. Gorkovaya, D. O. Ivanov, Y. S. Aleksandrovich

**Affiliations:** Department of Psychosomatics and Psychotherapy, Saint Petersburg State Pediatric Medical University, Saint Petersburg, Russian Federation

## Abstract

**Introduction:**

The COVID-19 pandemic required widespread adaptation to new environmental stressors, affecting the mental and physical health of diverse populations. A critical factor in this adaptation process is self-regulation. Self-regulation behaviour styles are stable patterns of voluntary action control that vary by individual and can influence responses to stress and adaptation challenges. In a pandemic context, these styles may influence the manifestation of asthenia.

**Objectives:**

This study investigates self-regulation behaviour styles and levels of asthenia in two distinct groups within the Russian population during the second wave of the COVID-19 pandemic: humanities students and HIV-positive patients. The study further examines relationships between self-regulation styles and asthenia within each group.

**Methods:**

Data collection was conducted from January to July 2021 through a specially developed Google form. The sample included 35 students from Russian universities specializing in the humanities and 59 HIV-positive patients. The study employed the “Behavioural Self-Regulation Style” questionnaire by V.I. Morosanova to measure self-regulation styles and profiles. To assess asthenia levels, the study used the MFI-20.

**Results:**

Self-Regulation Levels: Among students, 20% displayed low levels of self-regulation, while 37% showed high levels. In the HIV-positive group, 10% exhibited low self-regulation, and 37% exhibited high self-regulation. The self-regulation behaviour styles between the two groups did not show statistically significant differences. Analysis of asthenia revealed no significant differences between the groups in general asthenia (students: M = 11.71 ± 1.69; patients: M = 12.00 ± 1.87; p > 0.05), reduced activity (M = 11.94 ± 1.71 vs. M = 12.00 ± 2.12; p > 0.05), decreased motivation (M = 11.31 ± 2.23 vs. M = 12.80 ± 3.19; p > 0.05), physical asthenia (M = 13.31 ± 1.51 vs. M = 13.00 ± 1.22; p > 0.05), and mental asthenia (M = 11.66 ± 1.71 vs. M = 10.80 ± 1.92; p > 0.05). Among students, general asthenia correlated positively with programming (rs = 0.336, p < 0.05).Within HIV-positive group, general asthenia was negatively correlated with performance appraisal (rs = -0.889, p < 0.05). Additionally, reduced activity was correlated with performance appraisal (rs = -0.889, p < 0.05). Physical asthenia were negatively associated with autonomy (rs = -0.949, p < 0.05) and overall self-regulation (rs = -1.000, p < 0.01).

**Conclusions:**

Students and patients with HIV did not differ in self-regulatory behaviour styles and levels of asthenia during the pandemic. Among students, general asthenia appears linked to an increase in behavioural programming, which may serve as a compensatory mechanism for resource mobilisation in the face of fatigue. Within the HIV-positive group, asthenia is strongly associated with reductions in self-regulation.

**Disclosure of Interest:**

None Declared

